# Roles of Proteoglycans and Glycosaminoglycans in Cancer Development and Progression

**DOI:** 10.3390/ijms21175983

**Published:** 2020-08-20

**Authors:** Jinfen Wei, Meiling Hu, Kaitang Huang, Shudai Lin, Hongli Du

**Affiliations:** School of Biology and Biological Engineering, South China University of Technology, Guangzhou 510640, China; 201810107408@mail.scut.edu.cn (J.W.); humeiling@scut.edu.cn (M.H.); huangkaitang@foxmail.com (K.H.); linsd@scut.edu.cn (S.L.)

**Keywords:** proteoglycans, glycosaminoglycans, extracellular matrix remodeling, tumor microenvironment, cancer progression

## Abstract

The extracellular matrix (ECM) spatiotemporally controls cell fate; however, dysregulation of ECM remodeling can lead to tumorigenesis and cancer development by providing favorable conditions for tumor cells. Proteoglycans (PGs) and glycosaminoglycans (GAGs) are the major macromolecules composing ECM. They influence both cell behavior and matrix properties through direct and indirect interactions with various cytokines, growth factors, cell surface receptors, adhesion molecules, enzymes, and glycoproteins within the ECM. The classical features of PGs/GAGs play well-known roles in cancer angiogenesis, proliferation, invasion, and metastasis. Several lines of evidence suggest that PGs/GAGs critically affect broader aspects in cancer initiation and the progression process, including regulation of cell metabolism, serving as a sensor of ECM’s mechanical properties, affecting immune supervision, and participating in therapeutic resistance to various forms of treatment. These functions may be implemented through the characteristics of PGs/GAGs as molecular bridges linking ECM and cells in cell-specific and context-specific manners within the tumor microenvironment (TME). In this review, we intend to present a comprehensive illustration of the ways in which PGs/GAGs participate in and regulate several aspects of tumorigenesis; we put forward a perspective regarding their effects as biomarkers or targets for diagnoses and therapeutic interventions.

## 1. Introduction

Recently, the increasingly appreciated roles of extracellular matrix (ECM) remodeling, especially its altered mechanics properties, have prompted investigations in cancer [1]. ECM organization is spatiotemporally regulated to control cell behavior and phenotype via complex and regular interactions with matrix molecules. Dysregulation of ECM remodeling contributes to cell fate mainly through changing rigidity and structure; this loss of tissue homeostasis has been linked to many cancer hallmarks including metabolic reprogramming [1], persistent growth signals [2], immunosuppression [3], angiogenesis [4,5], tumor invasion [6] and metastasis [7]. In addition, ECM remodeling-related molecules have also been linked to drug resistance in cancer treatment [8] and have been indicated as diagnosis biomarkers to predict cancer outcome [9]. Overall, the broader roles of ECM remodeling with its properties in cancer initiation and progression should be taken seriously.

To explore how the disturbance of ECM remodeling affect cancer, it is essential to unravel the unique composition and status of its cytoplasmic matrix. As a dynamic structure, the function of ECM in cancer progression is primarily through its components, the deregulated feedback between cellular components and their local TEM, and biomechanical and biochemical properties of the matrix. The unbalance between degradation and secretion of ECM, arranged by ECM-modifying cells and ECM proteases, is responsible for disordered ECM remodeling. For example, cancer-associated fibroblasts (CAF) is observed in cancer surrounding matrix and accelerate cell migration and invasion through protease-mediated ECM remodeling [10]. Matrix metalloproteinases (MMPs) reshape the ECM to promote tumor development through various mechanisms including collagen degradation. MMPs stimulate cancer cell proliferation through denaturation of fibrillar collagen in melanoma [11]. Proteoglycans (PGs) and glycosaminoglycans (GAGs) are ubiquitous structural and functional components of ECM. Disorganized expression and distribution of PGs/GAGs lead to dysfunctional ECM and matrix structure collapse [12]. Based on structural features of PGs, composed of a core protein and GAGs, PGs bind several molecules, including various growth factors, adhesive factors, and cytokines, to modulate cancer progression [13]. These effects in cancer may be specific to individual PGs or the complex network between PGs and multiple ECM factors. Any changes in the balance of PGs, GAGs or ECM factors could reflect disorders of the local TME. Thus, understanding the mechanisms of overall or individual PGs/GAGs in the corresponding ECM are vital to understand the theory of driving tumor progression. These elements in in blood or urine represent the most encouraging biomarkers for cancer diagnosis and prognosis.

This review focuses on the importance and novelty of PGs/GAGs with their unique properties in cancer progression at several levels: regulation of cellular metabolism, regulating cell growth and apoptosis signals, managing cell invasion, and participating in immune destruction. We describe the molecular components, classification, metabolic enzymes, and signaling pathways involved in PGs/GAGs and summarize the interactions between PGs/GAGs and TME as well as the ways by which they influence tumor progression and govern responses of tumor cells to therapy. We discuss the potential effects of GAGs/PGs for diagnosis and therapy to prevent or control human cancer. Finally, we highlight challenges that require further investigation and the techniques and model systems that may facilitate this field.

## 2. Multiple Characteristics of PGs and GAGs

Because PGs are composed of a core of proteins covalently linked to GAGs, understanding the structural characteristics and classification of PGs/GAGs is significant for the exploration of the primary biological function in cancer development. Major GAGs include chondroitin sulfate (CS), dermatan sulfate (DS), keratan sulfate (KS), heparan sulfate (HS), and hyaluronic acid (HA) [14]. Except for HA, GAGs can be sulfated and linked to a core protein. For example, to CS is bound aggrecan, neurocan, veriscan, and brevican [15]. PGs are classified according to the chain characteristics of GAGs as well their distribution patterns in human cancer (Table 1).

Generally, the function of PGs/GAGs in cancer development may partly depend on concentration, spatial extension distribution, and temporal duration. Thus, the disordered metabolism of PGs/GAGs is associated with cancer malignancy; for example, the CS synthesis process was more active in several cancer types compared with normal tissues in our earlier study [16]. This was recently reviewed, and it was suggested that pharmacological treatment is a potential option for cancer therapy by targeting the chemical structure, synthesis, and degradation processes of PGs/GAGs [15,17,18]. Here, we focus on the important enzymes involved in the degradation processes and summarize the biomarkers associated with these enzymes in cancer (Table 2). Prominent examples are heparanase (HPSE) in degrading HS, hyaluronidase (HYAL) in degrading HA, and MMPs in cleaving PGs. Given the functional diversity of HS chains, such as regulation of the activity of cytokines and growth factors, HPSE-mediated degradation of HS is key to tumor development. Increased HPSE promotes tumor invasion [19] and angiogenesis in pancreatic neuroendocrine tumors and pediatric cancer [20]. Although some HPSE inhibitors have been used in clinical trials, the effects have been unsatisfactory. The main reason may be that inhibitors also target other molecules, causing off-target phenomena [21]. HYAL degrades HA into fragments of different sizes to participate in cancer development, as molecular signals and the level of HYAL expression is often disturbed in cancers. The overexpression of HYAL1 [22] and HYAL2 [23] is observed at the beginning of invasion in human breast cancer and is associated with cancer progression. Combining nanovaccine with HYAL has offered a simple and efficient strategy for inducing a powerful anti-tumor effect, indicating it is a promising method in cancer immunotherapy [24]. The MMPs are a family of proteolytic enzymes that degrade many ECM components, including PGs, and display disordered expression in various cancer types, serving as biomarkers for diagnosis and prognosis [25]. In addition to their roles in cell invasion, MMPs play a prominent function in cell proliferation, survival, immune response, and angiogenesis [26]. Once activated, MMP-9, secreted by tumor-associated neutrophils (TANs), cleaves various PGs in the extracellular space, destroying the existing tissue structure, releasing angiogenesis-related factors, and opening up space for expanded tumors to enhance angiogenesis [27]. MMP-9 also protects insulin-like growth factor (IFG) from inactivation via insulin-like growth factor binding protein (IGFBP), thereby providing growth signals for neighboring tumor cells [28]. MMP inhibitors (MMPIs) have been synthesized and tested in various cancer types over the last 20 years [29]; however, trials have been unsuccessful in reducing tumor burden or improving overall survival. We propose that these enzymes in cancer are not up or downregulated, but that there are also spatio-temporal changes throughout cancer development. We suggest that the first step should be to perform trials in early cancers, including measuring expression levels in the early stage. Then, developing drugs to target these enzymes will be very important and challenge investigations. The interaction between PGs/GAGs and fibrillar collagen contributes to fibrosis and affects ECM stiffness, [30] thereby influencing the progression of disease. Collagen XIII, a transmembrane protein, interacts with other ECM molecules, such as perlecan [31], and enhances angiogenesis by activating the JNK pathway and mediating ECM remodeling [32]. However, the roles of PGs/GAGs in cancer-associated ECM stiffness will be another significant research subject.

**Table 1:** Major PGs grouped according to their location, interaction signals, and their predominant GAGs during tumorigenesis.

Specific PG	GAGs	Interaction Signals/Patways	Implication in Cancer Types
**Hyalectans**			
Versican (up)	CS/DS	Snail/PAPSS2	Enhances cell migration and metastasis in breast cancer [33].
		FOXA2-VCAN	Promotes cancer growth and metastasis in ovarian cancer [34].
		/	Reduces tumor-infiltrating CD8-positive T-cells in cervical cancer [35].
		TGF-β/NF-κB signaling pathway	Promotes cancer invasion in ovarian cancer [36].
Aggrecan	CS/KS	/	Not studied in cancer.
Neurocan (up)	CS	/	Promotes malignant phenotypes in neuroblastoma [37].
Brevican (up)	CS	/ BEHAB-brevican	Promotes cell motility in glioma cancer [38]. Increases aggressiveness in gliomas cancer [39].
**Small Leucine-Rich PGs**			
Biglycan (up)	CS/DS	/	Enhances migration and invasion in endometrial cancer [40] and bladder cancer [41].
		Inducing integrin-β1	Promotes invasiveness in melanoma [42].
		NF-κB signaling	Promotes chemotherapy resistance in colon cancer [43].
		VEGF	Promotes angiogenesis in colon cancer [44].
		FAK signaling pathway	Enhances invasion in gastric cancer [45].
Decorin (down)	CS/DS	E-cadherin	Inhibits growth and migration in colon cancer [46].
		PDCD4/microRNA-21	Boosts inflammatory activity and suppresses tumor growth in blood cancer [47].
		TGF-β pathway	Inhibits invasion and metastasis in non-small cell lung cancer [48].
Lumican (down)	KS	AMPK signaling pathway	Augments chemotherapy cytotoxicity in pancreatic cancer [49].
		/	Restrains cancer growth in pancreatic cancer [50].
		/	Potentiates immunotherapy in breast cancer and melanoma [51].
		AMPK/HIFIA	Promotes cell apoptosis and inhibits cancer growth in pancreatic cancer [52].
		p120-catenin	Restrains cell invasion in lung cancer [53].
Lumican (up)		Autocrine regulatory	Promotes metastasis in lung cancer [54].
Fibromodulin (up)	KS	/	Promotes cancer progression in colonic cancer [55].
		TGF-β1 pathway	Promotes cancer migration in glioma cancer [56].
**Basement Membrane PGs**			
Perlecan (down)	HS/CS	/	Reduces metastatic burden in breast and lung cancer [57].
		FAK signaling	Abrogates cancer cell invasion and progression in prostate cancer [58].
Perlecan (up)		P53	Promotes metastatic and chemoresistance in pancreatic cancer [59].
Agrin (up)	HS	VEGFR2	Promotes tumor angiogenesis in liver cancer [60].
		MuSK signaling	Drives tumorigenesis in liver [61].
**Cell Surface PGs**			
Syndecan1 (up)	HS/CS/DS	KRAS	Fuels cell growth and promotes cancer development in pancreatic cancer [62].
		Notch and EGFR signaling pathways	Modulates cancer stem cell in breast cancer [63].
Glypican1 (up)	HS	/	Promotes angiogenesis and metastasis in varieties cancers [64].
		G2/M phase cell cycle	Promotes progression in cervical cancer [65].
Glypican 3 (up)	HS	Wnt signaling	Promotes tumor growth in hepatocellular carcinoma [66].
Glypican 5 (down)	HS	Wnt/β-catenin signaling	Inhibits tumor growth in lung cancer [67].
		G1/S phase arrest	Suppresses metastasis in non-small cell lung cancer [68].
**Intracellular PGs**			
Serglycin (up)	CS	CD44	Promotes cell aggressiveness in lung cancer [69].
		/	Promotes metastasis in nasopharyngeal cancer [70].
		IL-8 signaling	Promotes cell aggressiveness in breast cancer [71].

Note: “up” and “down” indicate that the specific PG is upregulated and downregulated, respectively, in cancer cells/tissues compared with normal ones in the corresponding studies. “/” indicates that the interaction signals or pathways are not shown in these studies.

## 3. GAGs and PGs: Connecting the Cell to the ECM

In addition to being major components of the ECM, PGs play roles in signal transduction by binding molecules within ECM, thereby affecting cancer progression. PGs’ core proteins interact with various receptors and glycoproteins including CD44 [69], EGFR [170], and VEGFR [171]. Binding with TGF-β, EGFR, HGFR, IGFR, and other growth factors, decorin weakens their downstream signals and inhibit tumor cell proliferation [172,173]. Unlike decorin, cell surface PGs are involved in promoting cell growth by interacting with growth factors and affecting proliferation signaling. As a sensor to the ECM mechanical properties, syndecan-4 binds to fibronectin to activate a series of signals, including the MAPK pathways, leading to cell proliferation and migration [174]. Syndecan-1, a coreceptor and cooperator with HGF [175] and HER2 [176], respectively, activates proliferative signals and improves cancer cell survival. Perlecan interacts with various cytokines including FGF and VEGF families through its core protein and the HS chains to affect angiogenesis and cell growth [177,178]. HSPG, harbored by CD43, enhances CXCL14 binding properties to glycoproteins, thereby leading to cell proliferation and tumor invasion in lung cancer [179]. CD43, a cancer-associated glycoprotein, is involved in various cancer types, where it performs several functions, including immune responses [180,181], cell proliferation [182], and cell growth and viability [183]. As other key macromolecules in ECM, alterations of glycoproteins, contributed by aberrant glycosylation patterns, are also observed in cancer. For more information about glycosylation in cancer please refer to the review in Reference [184].

**Figure 1:**
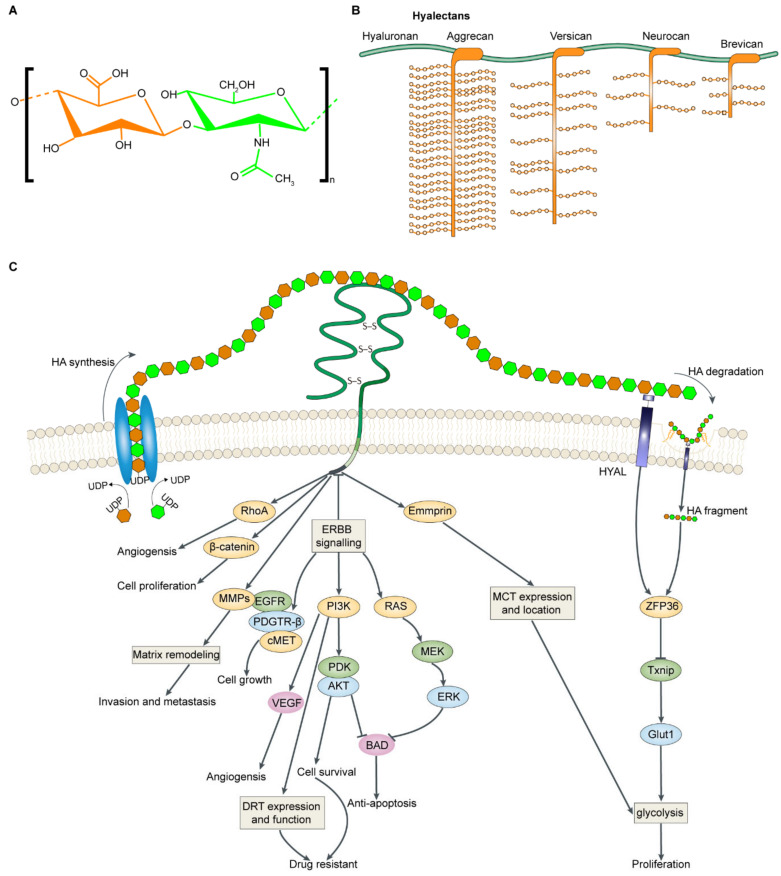
HA in tumor development. (**A**) Structure of HA. (**B**) The interactions between HA and hyalectans (aggrecan, versican, neurocan, and brevican) in the ECM. (**C**) The synthesis, degradation process, and roles in cancer progression of HA. HA is synthesized by HAS1, HAS2 and HAS3 in cell membrane. CD44 binds to HA to transduce signaling and its downstream effectors. HA-CD44 binds RhoGTPases and β-catenin to induce angiogenesis and proliferation. HA-CD44 stimulates MMP production and MMP presentation on cell surfaces and thus leads to cell invasiveness and metastasis. HA-CD44 induces ERBB2 activity to active PI3K/AKT, RAS/ERK, and EGFR signals. PI3K can activate EGFR /PDGTR/cMET to promote cell growth. PI3K signaling regulates DRT expression and function also phosphorylates AKT to activate cell-survival signaling thus inducing drug resistance. PI3K/AKT and RAS/ERK signal phosphorylate, the pro-apoptotic factor BAD, to inactivate it. HA-CD44 interacts with emmprin which locate MCTs on the cell membrane to increase the release of lactate and glycolysis. HA is degraded by HYAL into smaller molecules. HA fragment signals through RTKs to activate ZFP36, causing TXNIP degradation, hindering TXNIP-mediated GLUT1-internalization, leading to more glucose transport and prompting glycolysis to motivate cancer cell proliferation. HAS: hyaluronan synthase; HA: hyaluronan; MMP: metalloproteinase; DRT: drug-resistance transporter; HYAL: hyaluronidase; RTKs: receptor tyrosine kinases.

GAG chains also bind several factors to affect cancer progression. Although HS and CS bind growth factors or chemokines to regulate corresponding functions [185], HA is the most studied and important GAGs that deserve attention. In this section, we focus on HA-binding factors and point to the key signals in Figure 1. Compared with other GAGs forming PG, the basic structure of HA is a large polysaccharide that can interact with various membrane receptors and cell surface glycoproteins, including CD44, HMMR, EMMPRIN, and LYVE-1, to control tumor cell fate. The interaction of HA-CD44 greatly influences the key functional status of tumor cells in various aspects including promoting cell proliferation and enhancing chemo resistance through regulating PI3K/Akt and MAPK signal pathways [186,187]. Additionally, blocking HA-CD44 interaction is implicated in P53-dependent apoptosis in human lung cancer cells [188]. Another HA receptor, LYVE-1, with a similar structure to CD44, is also associated with chemoresistance for virus-mediated lymphoma [189]. LYVE-1 serves crucial roles in activating signal transduction pathways, including PIAK/Akt and NF-κB that regulate apoptosis and cell survival [190,191]. Based on these findings, HA appears to participate critically in tumor progression by interacting with receptors and acting on intracellular signaling pathways. Recent research has indicated that mechanical properties affect cell behavior in several circumstances including driving invasion and metastasis of cancer cells [10]. The structural and functional alterations in ECM composition define tumor-promoting physical and biomechanical properties of the ECM, such as stiffness, thereby allowing cancer cells to respond to mechanical stimuli and find escape mechanisms in the surrounding TME [192]. Cancer cells interact with and respond to these pressures, including ECM stiffness, through transmembrane PGs, such as syndecan, that induce MAPK signals to deal with these stresses and lead to cell survival and migration [174]. GAGs contribute ECM stiffening by regulating collagen structures, thereby controlling tumor growth [193]. Taken together, the data suggest that disordered interactions between PGs/GAGs and various molecules with altered expression and distribution are at least partially responsible for reconstructed ECM in the TME. It is suggested that combined targeting PGs/GAGs with their connected signaling pathways is an effective approach to improve therapeutic efficacy. This phenomenon can be demonstrated, for example, by co-treatment with lumican which enhances the cytotoxicity of chemotherapy by blocking AMPK signaling pathways in several models of pancreatic ductal cancer (PDAC) [49].

## 4. Dysregulation of GAGs/PGs in Cancer Progression

Several studies suggest that GAGs/PGs have important roles in cancer development, including regulation of metabolic patterns, angiogenesis and distant metastases, and treatment resistance [194], even playing roles in adaptive responses to stresses such as hypoxia and acidosis in the TME [195].

### 4.1. Metabolic Reprogramming

Metabolic deregulation is a hallmark of cancer. Increased glycolysis provides energy for cell growth and its intermediate products for macromolecular synthesis to cells, thereby enhancing malignancy [196]. Metabolic activities are affected by many biological properties, including ECM remodeling, and this effect is beginning to be deciphered in recent studies. The persistence of high glycolytic rates is shown in cancer to resist the pressure brought by the continually altered mechanical character of ECM [1]. Increased ECM stiffness contributed by CAFs enhances cell glutaminolysis to support the metabolic needs of tumor proliferation in several cancer types [197]. The close links between metabolic reprogramming and specific perturbations in PGs/GAGs, especially HA, are beginning to be established (Figure 1). HA fragments broken down by HYAL signal through receptor tyrosine kinases (PTKs) to induce downstream signaling, leading to increased glucose transport and induction of glycolysis to accelerate migration of cancer cells [198]. In addition to extensive conventional effects in malignancy, the interactions of HA-CD44 also play cooperative roles in tumor glycolysis. Antagonists of HA-CD44 interactions rapidly inhibit lactate production in breast cancer cells. Emmprin, a member of the immunoglobulin family, interacts with monocarboxylate transporters (MCTs) at the plasma membrane to increase glycolysis and enhance malignancy [199]. Similarly, another study suggested that interactions among HA, CD44, and emmprin contribute to cancer cells with glycolytic phenotype and other malignant properties [200]. In addition to HA, increased levels of PGs can also regulate glycolysis to influence cancer progression, these include lumican, an anti-proliferative PG that inhibits cell glycolysis by suppressing EGFR and its downstream signals in PDAC [104]. The CD36-glypcian 4 interaction inhibits the β-catenin-c-MYC signaling axis mediating glycolysis to repress colorectal cancer by ubiquitination of GPC4 [201].

We conclude that the precise signaling events between PGsß/GAGs and metabolism depend on the direct interaction with effectors, such as membrane glycoprotein, and these links between GAG chains and glucose metabolism might further explain that the matrix is a way to coordinate these interdependent processes. ECM remodeling caused by PG/GAG disorders serves as a key extrinsic site of cell metabolic regulation; therefore, we propose that PGs/GAGs can be considerable therapeutic targets in ECM to normalize glucose metabolic disorders responsible for cancer progression.

### 4.2. Tumor Cell Proliferation and Growth

Persistent cell proliferation signaling is another cancer hallmark. PGs/GAGs are involved in regulating tumor cell proliferation through modulating growth factors and signals. CS inhibits PTEN, a cancer suppressor, to activate melanoma cell proliferation [202]. Abnormal abundance of KS is highly associated with accelerated proliferation in tumors such as lymphoma [203], astrocytic tumors [204], and glioblastoma [205]. Growth signals are activated by sulfated KS that induces the MAPK and PI3K pathways in lymphoma cells [203]. HS, presenting on cell surfaces and in matrix, is involved in modulating cell growth. In one study, HS acted as a receptor for a growth-related ligand to promote cancer growth [206]. We suggest that, in the future, combining anti-GAGs drugs with proliferative inhibitors may represent an attractive approach in clinical treatments for cancer therapy.

Via interactions with signaling molecules, PGs cooperate with ECM proteins and cell proliferation-related signaling events, including NF-κB and EGFR signaling pathways, to regulate tumor growth. Versican is a large CSPG involved in regulating cell proliferation in several cancer types. The matrix versican secreted by stroma cells promoted cancer cell proliferation by interacting with HA-CD44 signals in ovarian cancer [36]. Biglycan knock-out led tumor cell apoptosis by decreasing cyclin A and cyclin D1 expression in colon cancer [207]. As mentioned above, lumican, an antiproliferative PG, inhibited cell growth potential in melanoma cells [208,209] and inhibited cell proliferation in animal models of melanoma [210] and pancreatic cancer [104]. Additional supplementation with lumican suppressed cell growth by binding to EGFR and, subsequently, blocked downstream pathways in pancreatic cancer [50]. Perlecan, another antiproliferative PG, bound growth factors and blocked tumor cell proliferation through both its GAG chains and core protein [211]. Stroma cell-derived perlecan slowed the proliferative potential, thereby reducing tumor burden in lung and breast cancer [57]. Cell surface PGs also participate in regulating cell proliferation [212]; this includes the syndecan family [213]. One study showed that syndecan-1 was critical for tumor presence and growth, serving as a KRAS activator, and this suggested that it was a potential therapeutic target in PDAC treatment [62]. Serglycin, the only intracellular PG, promoted tumor cell growth through interacting with CD44, and it was found that combining targeting serglycin and CD44 could be an effective therapy [69]. Based on a growing body of studies, specific PGs with different core protein and GAG chain can be used as tumor suppressor or oncoprotein to influence cell growth, providing promising therapeutic strategies for cancer.

### 4.3. Angiogenesis

Sustained angiogenesis is another hallmark of cancer, and angiogenesis allows tumors to grow due to the access to an ample nutrient supply which can be regulated by several angiogenic factors including VEGF and FGF-2 [214]. The significant roles of ECM remodeling in tumor angiogenesis have been discussed and highlighted in a previous review [215]. We concentrate on the current knowledge regarding how PGs/GAGs regulate angiogenesis in cancer. HA is the key link between ECM-cancer-angiogenesis [216]. HA overproduction within the ECM rapidly develops aggressive breast carcinomas in which cancer cells engage in more vascularization [217]. Generally, HA interacts with versican, the most abundant type of PG, to affect angiogenesis [218]. Matrix versican, secreted by stromal cells, promoted cancer growth by inducing angiogenesis in lung cancer [219]. Biglycan contributes to tumor growth partly due to the fact of its capability to stimulate inflammation-related angiogenesis. For example, high levels of biglycan triggered angiogenesis by upregulation of VEGF and the TLR signaling pathway in colon cancer [44] and gastric cancer [220]. As another effective regulator in angiogenesis, perlecan promotes angiogenesis by activating vascular-related factors based on its HS chain [221] and by protecting angiogenic factors from specific inhibitory biological functions such as proteolysis [222]. A high level of matrix agrin is vital for angiogenesis; this was confirmed by a study in which agrin stimulated angiogenesis by upregulating VEGFR2 levels in liver cancer [60]. Other PGs, such as glypicans, also play a part in angiogenesis by regulation of angiogenic factors. Endothelial cell-derived glypican-1 promoted angiogenesis by regulating FGF2 and VEGFA in pancreatic cancer [223]. Studying the specific mechanisms of the interaction between PG and these angiogenesis factors provides more possibilities for us to choose appropriate inhibitors to block angiogenesis in cancer treatment.

### 4.4. Tumor Cell Invasion and Metastasis

Cell invasion and metastasis is associated with tumor recurrence, poor survival, and mortality. ECM remodeling is a major event that promotes cancer invasion by creating suitable conditions including inducing podosome biogenesis for facilitating cell invasion in breast cancer [224]. PGs/GAGs regulate cancer metastasis in several aspects by a specific PG/GAG or collective activity of PGs/GAGs. A research method developed to calculate the scores defining measured GAG abundance robustly predicted cancer cell metastasis in renal cancer [225]. Alteration in one individual GAG also led to tumor metastasis; for example, elevated HS biosynthesis in a matrix highly correlated with cell migration [226]. Conventionally, the role of HS in cancer metastasis is mainly due to the fact of its interaction with growth factors as well as its regulation of the epithelial-to-mesenchymal transition (EMT) which is the first signal of metastasis [227]. In addition to conventional functions, some novel characteristics of HS in cancer metastasis have also been reported. HSPG activates cancer’s metastatic potential to promote tumor progression by acting as a stress sensor to adapt the cellular response to hypoxic stress within the TME [195]. Studies showed that expression, abundance, structural changes, cell-derived sources, and location of PGs influence cancer metastasis. Sulfation of glycan promoted tumor metastasis in lung cancer [228]. High expression of versican enhanced cell migration and metastasis in several breast cancer cell lines [33]. Myeloid cell-derived versican, a macrophage activator, facilitated cancer metastatic growth in lung cancer [229]. Fibroblast-derived syndecan-1 is vital to promote invasion and metastasis in breast cancer cells [230]. Serglycin is an intracellular PG that aggregates with CD44 to promote lung cancer cell migration by triggering the CD44/NF-κB/CLDN1 [69] and CD44/Rho/Src/FAK axes [231]. These significant studies allow us to further understand the cell source, abundance, location, and expression levels of PG/GAGs and to explore more deeply the mechanisms of regulatory interactions between PGs/GAGs and ECM signaling. These will help us identify better treatments to block cancer metastasis in a timely fashion, thereby improving survival rates.

### 4.5. Immune Surveillance

Tumor progression is also regulated and supervised by immune infiltration (TIL) and immune cell ability for eliminating tumors. Immune surveillance is partly influenced by dynamic changes of ECM, as is illustrated by a study in which constituents’ alteration of ECM promoted tumor survival and affected the motility of T immune cells in melanoma [7]. Increased ECM stiffness contributed by high collagen density in breast cancer ECM reduced T-cell cytotoxic activity and supported cancer progression [232]. According to previous studies, the role of PGs/GAGs in regulating the supervisory function of immune cells depends on their particular molecular structure and the intimate connection between PGs/GAGs and immune signaling; for example, changing the glycoprotein form of various immune-related cytokines enhanced immune escape in several types of solid tumors [233]. GAGs are major factors in tumor immunity, for example, HS as a candidate for therapeutic cancer vaccination against various malignancies [234]. The interaction of HA fragments with CD44 in the ECM weakened the anti-tumor ability of cytotoxic T lymphocytes by reducing Fas-mediated apoptosis in lung cancer [235]. Tumor-derived HA fragments contributed to immune escape by “instigating” dendritic cells in a special pattern inducing apoptosis of autologous T-cells in several tumors [233]. Recently, HA-based hydrogel has been engineered to T-cell activation by presenting the two stimulatory signals [236].

Versican, a large matrix PG with immunoregulatory activity highly expressed in the TME matrix, is known to reduce the tumor-infiltrating level (TIL) of CD8+ T-cells and promote cell migration in cervical cancer [35]. It is expected that removing versican could suppress its cancer-promoting effect; for example, increasing versican proteolysis enhances the CD8+ T-cell infiltration in colorectal cancer [237]. Acting as TLR ligand, a high level of versican also regulates the immunosuppressive ability of myeloid-derived suppressor cells (MDSCs) to enhance the immune escape [238]. These studies provide a way for investigating versican as a novel immune biomarker in solid cancers. Intriguingly, decorin reduced the abundance of anti-inflammatory molecules and increased proinflammatory molecules, thereby boosting the immune response and reducing tumor growth. Decorin signaling suppressed tumor growth by stimulating PDCD4, shifting the immune response toward a proinflammatory phenotype in a tumor xenograft model [47]. In addition, to direct immunotherapy applications, considering the influences of the environment on treatment effect is vital to studying how to apply combination targets to immune stimulation in the future. Therefore, finding a reasonable combined targeting strategy will not only improve immune responses and avoid immune escape but will also block cancer progression directly.

## 5. Clinical Features

### 5.1. GAGs and GPs in Diagnosis and Prognosis

In addition to the critical roles in malignancies at multiple aspects described above, PGs/GAGs also have value for clinical diagnosis and prognosis in various cancer types. Because GAGs play roles in the ECM, researchers supposed that alteration of their abundances would reflect changes in body fluids, i.e., the blood and urine of cancer patients, e.g., levels of GAGs in plasma predicted the tumor metastatic capacity in renal cancer [225]. High levels of KS appeared in female genital cancer suggesting that KS might be a biomarker for diagnosis of this cancer [239]. High levels of CS showed prognostic capacity in breast [128] and ovarian cancers [130]. High expression levels of HS signaled poor prognosis in patients with gastric carcinoma [131]. Higher levels of HA were associated with poor survival in patients with breast cancer [133], acute myeloid leukemia [134], and prostate cancer [132]. Elevated serum levels of HA can be a diagnostic biomarker for patients with prostate cancer [132], upper gastrointestinal cancers [136], and mesothelioma [139]. The diagnosis and prognosis roles of HA in other cancer types are presented in Table 2.

PGs are prominent molecules in tumor diagnosis. The increased versican levels in cancer patients were shown to diagnose the occurrence of epithelial ovarian cancer [75] and multiple myeloma [76]. Elevated levels of glypican-1 were associated with poor survival in patients with PDAC [117]. Because glypican-1 is overexpressed in several cancer types [116,117], it can be a biomarker for detection of prostate cancer [115] in urine and for the dissemination of glioblastoma [119]. The abundance of glypican-1 positively correlates with disease severity in patients, whether they have received surgical treatment or not, suggesting that glypican is a surgery-independent, inherent diagnostic biomarker of pancreatic cancer [240]. Other studies relevant to prognosis and diagnostic capabilities of PGs/GAGs are summarized in Table 2. All these studies provide possible clinical values for early detection and prognosis of PGs/GAGs, especially those detected in urine or blood, and help design potential cancer treatments. Therefore, acceleration of the development of research tools in detection of PGs/GAGs, especially determination whether disordered PGs/GAGs can be secreted into the blood or excreted into the urine, will make it easier to detect cancer in early stages and will represent the most valuable biomarkers for cancer diagnosis.

### 5.2. GAGs and PGs in Cancer Treatment

In addition to their diagnostic and prognostic capabilities, PGs/GAGs play roles in therapy resistance in various therapeutic processes and are used as effective therapeutic targets for inhibition of cancer progression. Accordingly, drugs have been produced targeting GAGs’ metabolic processes including synthesis, degradation, and related enzymes as well as targeting their specific structures [241]. High levels of HA in the TME are related to more severe and advanced cancer states [242,243], reflected in promoting tumor progression and participating in therapeutic resistance. The role of HA in drug resistance is contributed by disordered interaction between HA–ECM factors in the TME. Large quantities of HA in ECM stimulate interactions with CD44 to cause chemoresistance in patients with head and neck cancer patients [244]. HA activates growth signals, such as the PI3K/Akt pathway, thus leading to chemotherapy resistance in breast cancer [245]. By activation of TGFβ/Smad2 signals and ECM, HA-HMMR conferred resistance to chemotherapy in gastric cancer [246]. Degrading HA or breaking down the interaction between HA and ECM molecules improved drug resistance and blocked cancer progression. Notably, HA depletion was shown to have very promising results in preclinical studies [247]. Because HYAL degrades HA, the activity and expression of HYAL can be adjusted as a choice to determine the concentration of HA in TME. Using HYAL to deplete HA leads NK cells to attach to a high HA matrix and significantly enhance trastuzumab or cetuximab-dependent cell-mediated cytotoxicity in breast cancer [248]. However, involvement in ECM remodeling leads HA to enhance the treatment effect. HA increased penetration by causing collagen degradation and further altering the dense extracellular space to looser space, thereby increasing the chemo-sensitivity in gallbladder cancer [249]. HA, as a material with special physiochemical properties, is now widely used as a promising tumor drug carrier; the application and prospects of HA for drug delivery has been reviewed in recent works [250,251]. Without controversy, HA plays a role in many aspects of cancer development. More studies are needed to research the control of the number and pattern of HA in the TME, to block the interaction between HA-related proteins and drug responses, all of which will bring great benefits to the cancer treatment.

As a tumor suppressor, lumican enhanced TIL and improved the ability of immune cells to eliminate tumors in several solid tumors [51]. By inhibiting tumor metastasis, adding exogenetic decorin inhibited the growth of prostate cancer [252]. However, most PGs contribute to malignancy by triggering therapeutic resistance; they could be the targets of cancer treatment. Through versican silencing, chemoresistance could be ameliorated, indicating that versican could be a potential therapeutic target in prostate cancer [253]. CSPG4-targeting, including immunotoxins and antibodies, improved therapeutic resistance by enhancing the effectiveness of photochemical internalization in breast cancer [254] and by inhibiting cell proliferation in melanoma patients [255]. To be more specific, PGs conferred resistance to tumor therapy by activating key signals such as activation of EGFR-AKT signaling by versican, inducing breast cancer self-renewal [256] and chemotherapy resistance [49], activating NF-κB signaling by biglycan to promote resistance to chemotherapy of colon cancer [43] and activating the EGFR pathway by syndecan-1 to lead to chemoresistance in colon cancer [257]. Circulating syndecan-1 contributed to chemotherapy resistance in prostate cancer [114]. For a more detailed presentation about the targeting of these molecules and the corresponding curative effect, the reader can read the recent comprehensive work presented in Reference [258].

As mentioned above, PGs/GAGs play important roles in tumor survival and therapeutic resistance, suggesting that PGs/GAGs can be attractive targets in cancer therapy. We propose that any changes in the ECM should be taken seriously, particularly those that may affect therapeutic effects. Therapies designed to combine targeting PGs/GAGs with anti-tumor therapy may be potential strategies, inhibiting tumor survival and improving patient outcome. For instance, targeting CD44 using HA-labeled nanoparticles overcame chemoresistance with a higher efficiency in an ovarian carcinoma PDX model [259].

## 6. Conclusions and Perspectives

The research regarding ECM remodeling has elucidated its role in cancer initiation and progression. Comprehensive understanding of the altered abundance, structure, localization of ECM components, and the interaction signals between cancer cells, stroma cells, and ECM may help us to find more valuable biomarkers in cancer diagnosis and prognosis as well as to develop effective potential therapies. Those studies about PGs, GAG chains, and PG/GAG-related enzymes and surface receptors have shown encouraging diagnostic and prognostic values in an impressive number of preclinical studies and experimental models. However, few examples for use of PGs or GAGs as biomarkers/targets in a clinical routine setting have been extensively used. Perhaps one reason is the complexity and diversity of PG/GAG structures, such that it is difficult to fully define their fragments in the local ECM of cancer patients. The other reason is that disruption of individual PGs or GAGs can influence their downstream cascades and other related-signaling functions, leading to disordered cross-interacting networks within the TME. Tumoral heterogeneity of ECM, including expression levels, distribution, and derived sources of PGs/GAGs, is another reason for the difficulty of applying therapies. It is still difficult to fully define the regulatory processes of PGs/GAGs through a variety of mechanisms in extremely dynamic remodeling of ECM. Therefore, finding molecular regulation rules of PGs/GAGs in complex networks as well as establishing appropriate models and developing research tools of PGs/GAGs will be essential to transform these findings into clinical applications for targeting PGs/GAGs in the future. For example, using a combination of cell biology, novel approaches in chemistry glycobiology, biomedical nanotechnology, single cell sequencing, and bioinformatics to visualize abundance, size, and location of PGs/GAGs in spatio-temporal states of specific cancer TME are the top priorities. Studying the mechanisms by which networks regulate PGs/GAGs and malignant cells and how they become deregulated in various cancer types, distinguishing beneficial or detrimental roles in cancer progression, and developing accuracy in fluid detection are all conducive to improving early diagnosis levels, preventing tumorigenesis, realizing precision medicine, suppressing cancer progression, and further improving patient survival.

## Figures and Tables

**Table 2:** The values of PGs/GAGs and related enzymes in pre-clinical and clinical studies of cancer.

Name	Samples	Potential Clinical Applications in Cancer
**PGs**		
Versican	Tissues (*n* = 84)	Prognosis for renal carcinoma [72]
	Tissues (*n* = 52, 62, 456/453, 89)	Prognosis for colon cancer [73,74]
	Blood (*n* = 27, 12, 31)	Diagnosis for epithelial ovarian cancer [75]
	Blood (*n* = 30)	Diagnosis for multiple myeloma [76]
	Tissues (*n* = 134/104)	Prognosis for gastric cancer [77,78]
	Tissues (*n* = 142/212)	Prognosis for non-small cell lung cancer [79,80]
	Tissues (*n* = 50, 19, 31)	Diagnosis for hepatocellular carcinoma [81]
	Tissues (*n* = 139)	Prognosis for oral squamous cell carcinoma [82]
	Tissues (*n* = 80/58)	Prognosis for breast cancer [83,84]
	Tissues (*n* = 111/111)	Prognosis for ovarian cancer [85,86]
	Tissues (*n* = 167)	Prognosis for endometrial cancer [87]
	Tissues (*n* = 43)	Prognosis for prostate cancer [88]
Biglycan	Tissues (*n* = 12,427)	Prognosis for prostate cancer [89]
	Tissues (*n* = 10)	Prognosis for gastric cancer [90]
	Tissues (*n* = 287)	Prognosis for colorectal cancer [91]
	Tissues (*n* = 62/170)	Diagnosis for esophageal adenocarcinoma [92,93]
	Tissues (*n* = 52)	Prognosis for endometrial cancer [94]
	Tissues (*n* = 53)	Prognosis for pancreatic adenocarcinoma [95]
Decorin	Tissues (*n* = 24)	Diagnosis for invasive ductal carcinoma [96]
	Tissues (*n* = 16)	Prognosis for malignant peripheral nerve sheath tumor [97]
	Tissues (*n* = 83, 6, 32, 145/64)	Prognosis for lung adenocarcinoma [98,99]
	Tissues (*n* = 16)	Prognosis for oral cancer and response to S-1 neoadjuvant chemotherapy [100]
	Plasma (*n* = 275)	Diagnosis for esophageal squamous cell carcinoma [101]
	Tissues (*n* = 140)	Prognosis for breast cancer [102]
Lumican	Tissues (*n* = 13)	Diagnosis for lung adenocarcinoma [103]
	Tissues (*n* = 131)	Prognosis for pancreatic cancer [104]
	Tissues (*n* = 158)	Prognosis for colorectal cancer [105]
	Tissues (*n* = 102)	Prognosis for lung adenocarcinoma and squamous cell carcinoma [106]
Perlecan	Tissues (*n* = 511)	Prognosis for oligodendroglioma [107]
Agrin	Tissues (*n* = 123)	Prognosis for oral cancer [108]
	Tissues (*n* = 86)	Prognosis for lung adenocarcinoma [109]
Collagen XVIII	Tissues (*n* = 105)	Prognosis for hepatocellular carcinoma [110]
	Tissues (*n* = 221/94)	Prognosis for lung carcinoma [111,112]
	Tissues (*n* = 118)	Prognosis for gastric carcinoma [113]
Syndecan1	Tissues (*n* = 111)	Prognosis for ovarian cancer [86]
	Serum (*n* = 75)	Prediction of docetaxel resistance in prostate cancer [114]
Syndecan 3	Blood (*n* = 27, 12, 31)	Diagnosis for epithelial ovarian cancer [75]
Glypican1	Urine (*n* = 203)	Diagnosis for prostate cancer [115]
	Tissues (*n* = 240/186/62)	Prognosis for pancreatic cancer [116,117,118]
	Tissues (*n* = 53)	Diagnosis dissemination and prognosis for glioblastomas [119]
	Tissues (*n* = 175)	Prognosis for esophageal squamous cell carcinoma [120]
Glypican3	Tissues (*n* = 2336)	Diagnosis for hepatocellular cancer [121]
	Blood (*n* = 85)	Prognosis for hepatocellular cancer [122]
	Tissues (*n* = 106)	Prognosis for pancreatic ductal cancer [123]
Glypican5	Tissues (*n* = 160)	Prognosis for prostate cancer [124]
	Tissues (*n* = 40/198)	Prognosis for lung adenocarcinoma [67,68]
Serglycin	Tissues (*n* = 112)	Prognosis for nasopharyngeal carcinoma [125]
	Tissues (*n* = 127)	Prognosis for hepatocellular carcinoma [126]
**GAGs**		
Plasma GAGs	Blood (*n* = 175)	Diagnosis and prognosis for renal cell cancer [127]
CS	Tissues (*n* = 130/169)	Prognosis for breast cancer [128]
	Tissues (*n* = 289/148)	Prognosis for ovarian cancer [129,130]
HS	Tissues (*n* = 162)	Prognosis for gastric carcinoma [131]
HA	Blood (*n* = 44)	Diagnosis and prognosis for prostate cancer [132]
	Blood (*n* = 212/334)	Prognosis for breast cancer [133]
	Serum (*n* = 51)	Prognosis for acute myeloid leukemia [134]
	Urine (*n* = 513)	Diagnosis for bladder cancer [135]
	Serum (*n* = 63)	Diagnosis for upper gastrointestinal cancers [136]
	Cytosol (*n* = 120)	Prognosis for colorectal cancer [137]
	Tissues (*n* = 46)/Sputum (*n* = 25)	Diagnosis and prognosis for lung cancer [138]
	Serum/pleural (*n* = 96)	Diagnosis and prognosis for malignant mesothelioma [139]
	Serum (*n* = 506)	Prognosis for liver cancer in hepatic resection [140]
	Tissues (*n* = 45)	Prognosis for nerve sheath tumor [141]
**Enzymes**		
HPSE	Tissues (*n* = 182)	Prognosis for glioma [142]
	Serum (*n* = 156)	Diagnosis for breast cancer [143]
	Serum (*n* = 177)	Diagnosis for ovarian cancer [144]
	Tissues (*n* = 81)	Prognosis for oral mucosal melanoma [145]
HYAL-1	Tissues (*n* = 407/178)	Prognosis for bladder cancer [146,147]
	Tissues (*n* = 70)	Prognosis for prostate cancer [148]
	Urine (*n* = 513)	Diagnosis for bladder cancer [149]
	Tissues (*n* = 34)	Prognosis for colorectal cancer [150]
HAS1	Tissues (*n* = 278)	Prognosis for breast cancer [151]
	Tissues (*n* = 31)	Prognosis for colon cancer [152]
	Tissues (*n* = 287)	Prognosis for prostate cancer [153]
HAS2	Tissues (*n* = 407)	Prognosis for bladder cancer [146]
HAS3	Tissues (*n* = 407)	Prognosis for bladder cancer [146]
	Tissues (*n* = 278)	Prognosis for breast cancer [151]
MMP2	Tissues (*n* = 1266)	Prognosis for oral cancer [154]
MMP3	Urinary (*n* = 70)	Diagnosis and prognosis for bladder cancer [155]
MMP9	Tissues (*n* = 1266)	Prognosis for oral cancer [154]
MMP14	Tissues (*n* = 456)	Prognosis for colorectal cancer [156]
MMP16	Tissues (*n* = 375)	Prognosis for gastric cancer [157]
**Effectors**		
RHAMM	Tissues (*n* = 383)	Prognosis for large cell lung cancer [158]
	Tissues (*n* = 64)	Prognosis for kidney cancer [159]
	Tissues (*n* = 33)	Prognosis for ovarian cancer [160]
	Tissues (*n* = 223)	Prognosis for colorectal cancer [161]
	Tissues (*n* = 89)	Prognosis for endometrial cancer [162]
	Tissues (*n* = 72)	Prognosis for B-cell chronic leukemia [163]
	Tissues (*n* = 210)	Prognosis for multiple myeloma [164]
CD44	Tissues (*n* = 64)	Prognosis for kidney cancer [159]
	Tissues (*n* = 94)	Prognosis for bladder cancer [165]
	Tissues (*n* = 145)	Prognosis for colorectal adenocarcinomas [166]
	Tissues (*n* = 158/333)	Prognosis for early gastric cancer [167,168]
	Tissues (*n* = 278)	Prognosis for breast cancer [169]

## Abbreviations

PGs	Proteoglycans
GAGs	Glycosaminoglycans
ECM	Extracellular matrix
CS	Chondroitin sulfate
DS	Dermatan sulfate
KS	Keratan sulfate
HS	Heparan sulfate
HA	Hyaluronic acid
EGFR	Epidermal growth factor receptor
HGFR	Hepatocyte growth factor receptor
IGFR	Insulin-like growth factor 1 receptor
HGF	Hepatocyte growth factor
HMMR	HA-mediated motility receptor
LYVE-1	Lymphatic vessel endothelial hyaluronic receptor-1
EMMPRIN	Extracellular matrix metalloproteinase inducer
RTKs	Receptor tyrosine kinases
TLR	Toll like receptor
